# Restricted versus Standard Maintenance Fluid Volume in Management of Transient Tachypnea of Newborn: A Clinical Trial

**Published:** 2014-10-01

**Authors:** Masoud Dehdashtian, Mohammad-Reza Aramesh, Arash Melekian, Mohammad-Hasan Aletayeb, Anahita Ghaemmaghami

**Affiliations:** Department of Pediatrics, Imam Khomeini Hospital, Ahvaz Jundishapur University of Medical Sciences, Ahvaz, Iran

**Keywords:** Transient Tachypnea of Newborn, Specific Gravity, Fluid, Newborns, Cesarean Section

## Abstract

***Objective:*** The incidence of Transient Tachypnea of Newborn (TTN) is higher in infants born by cesarean section than with  vaginal delivery. Treatment of transient tachypnea of newborn is supportive. The purpose of this study was to assess the effect of restricted fluid volume intake on the course of respiratory distress in patients with TTN.

***Methods:*** This is a quasi-experimental clinical trial of 83 neonates diagnosed with TTN admitted to a neonatal intensive care unit in south west Iran. In this study the effect of restriction of maintenance fluid volume in the course of respiratory distress in newborns with transient tachypnea was assessed.

***Findings:*** In the standard fluid volume intake group 18 (42.8%) cases needed nasal continuous positive airway pressure (NCPAP) and one (2.38%) case mechanical ventilation, and in restricted fluid volume intake group 13 (32.5%) cases needed NCPAP and two (5%) cases mechanical ventilation. 54.82% of cases were supported with oxyhood in the standard fluid volume and 62.5% in the restricted fluid volume intake group. Differences in duration of the needed NCPAP and oxygen hood between the two groups were significant. Fluid restriction had no adverse effect on the urine specific gravity or weight loss of the studied newborns.

***Conclusion:*** Limited fluid administered to newborns with transient tachypnea of newborn is safe and resulted in shorter duration of respiratory support.

## Introduction

Cesarean section rate has increased in Iran and most regions of the world^[^^1^^-^^3^^]^. Increased childbearing age, obstetricians’ ethical consideration and fear of the risks of vaginal delivery are the common causes for cesarean delivery^[^^4^^]^. Almost 90% of women with a previous cesarean section have a repeated cesarean delivery^[^^5^^,^^6^^]^. About 6% of elective cesarean sections in the United States are performed in response to maternal request^[^^7^^]^. Several studies have reported the higher incidence of significant respiratory distress in infants born by cesarean section^[^^8^^-^^11^^]^. Fetal lung contains approximately 40 ml/kg body weight liquid during late pregnancy^[^^12^^]^. For effective gas exchange to occur, the lung should be cleared of excess fluid. The failure of this event has been implicated in several newborn diseases, including transient tachypnea of newborn (TTN) and respiratory distress syndrome (RDS)^[^^13^^,^^14^^]^. 

 TTN is the most common cause of early respiratory distress in term and late preterm infants because of delayed reabsorption of the fetal lung fluid^[^^15^^]^. Conventionally the signs of TTN resolve within the first 72-96 hours of life^[^^16^^]^. However, there are several reports that TTN may be complicated with persistent pulmonary hypertension of newborn^[^^17^^,^^18^^]^. Treatment of TTN is supportive^[^^16^^]^. Traditionally the infants receive 60, 80 and 100 ml/kg/day fluid at first, second and third day of life, respectively. Considering that the volume of breastfeeding during the first three days of life is between 2-20 ml in each feeding and the average of breast milk is about 100 ml in the first 24 hours^[^^19^^]^, we conducted a quasi-experimental clinical trial study to examine the effect of restriction of fluid intake on the clinical course of neonates with TTN.

## Subjects and Methods

This quasi-experimental clinical trial study was performed at Neonatal Intensive Care Unit of Imam Khomeini Hospital, a teaching tertiary care center in Ahvaz, the capital of Khuzestan province of Iran with 36 beds. This study was approved by the Ethics Committees of Ahvaz Jundishapur University of Medical Sciences and registered with the Iranian Registry of Clinical Trials (IRCT2012111111432N1). 

 Newborns between 34-0/7 and 41-6/7 weeks gestational age with a diagnosis of TTN according to the criteria of Rawlings and Smith^[^^20^^]^, were eligible for inclusion in this study. Neonates with congenital anomalies, confirmed sepsis [WBC <5000/mm^3^, immature-to-total neutrophil (I:T) ratio >0.2, positive CRP, or positive blood culture), respiratory distress syndrome (reticulogranular pattern and airbroncho-gram in chest radiography and needing intratracheal surfactant), meconium aspiration, intrauterine growth retardation, birth asphyxia, serum sodium level ≥150 mg%, BUN >20 mg%, creatinine >1 mg%, and urine output less than 0.5 ml/kg/hour in the course of study^[^^21^^]^ were excluded. Patients were hospitalized at our neonatal intensive care unit from April 20, 2012 through May 13, 2013. Informed consent was obtained from patients’ fathers.

 Initially, the patients received fluid 60 ml/kg/day or 80 ml/kg/day on the first day of life. The fluid volume increased by 20 ml/kg daily up to a maximum of 100, or 120 ml/kg/d, if they were in an incubator or under a radiant warmer (control group). After completion of sample size of the control group, the fluid intake strategy was changed to 40 ml/kg/day or 60 ml/kg/day on the first day of life and increased by 20 ml/kg daily up to a maximum of 80, or 100 ml/kg/d, if they were in an incubator or under a radiant warmer (intervention group). 

 Sodium chloride 3 meq/kg/d and potassium chloride 2 meq/kg/d was added to the fluid from the second day of life. By initiation of enteral feeding intravenous fluid was reduced amounting to enteral feeding milliliter by milliliter. 

**Fig. 1:**
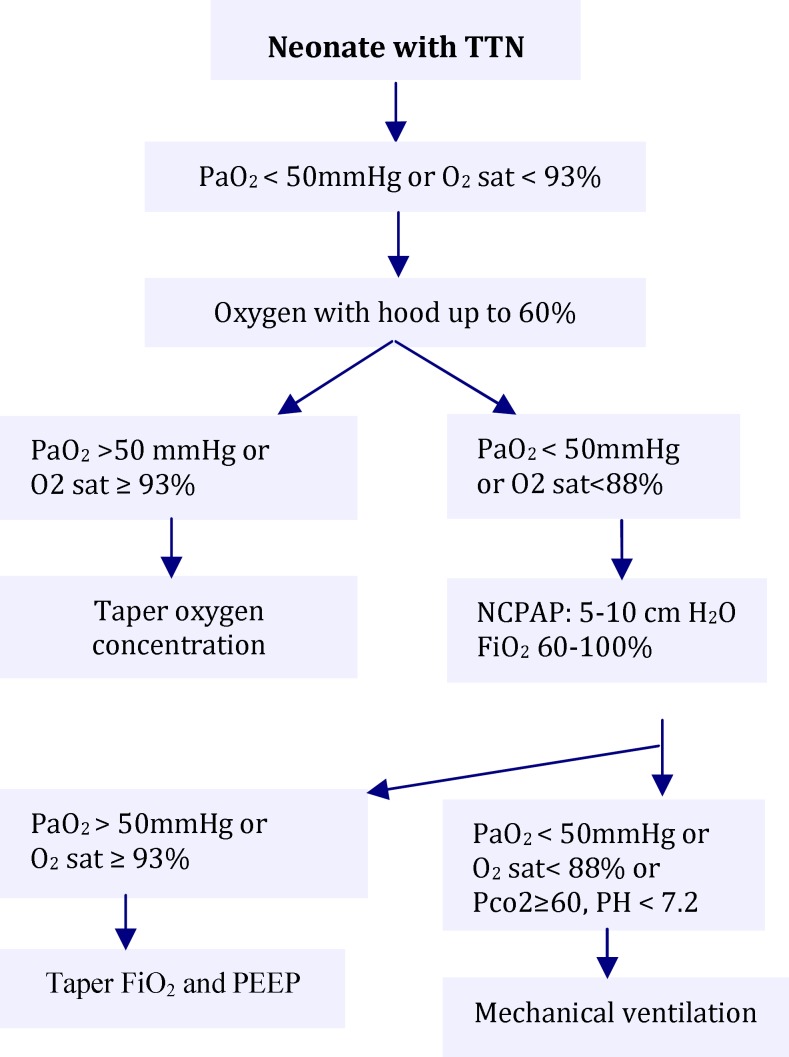
Approach to neonate with transient tachypnea of newborn (TTN) in this study

 Daily serum sodium, potassium, BUN, creatinine and twice daily urine specific gravity were measured in both groups. Patients, their families and medical staff were blinded to study. Contemporary urine specific gravity and weight was measured in 53 well baby newborn infants. Because well baby newborns were discharged at the second day of life, we could measure their weight and urine specific gravity only at the first and second day of life. Respiratory support was initiated in patients with oxygen saturation less than 93% or PaO_2_ 50 mmHg. The purpose of the treatment was to achieve oxygen saturation between 88% and 93% (Fig. 1). 

 Patients' safety was assessed by daily determination of serum sodium, potassium, glucose, BUN, creatinine and urine output. Urine specific gravity was measured twice daily. Patients’ clinical course was followed by measuring the arterial blood gas and employing a pulse oxymeter. The goal of the study was to determine the effect of fluid restriction on duration of need to respiratory support in newborns with TTN. Based on the study by Stroustrop et al, and using a two-tailed type I error rate of 0.05 and a power of 90%, sample size of 40 patients in each group was calculated. 

*Statistical analysis: *Comparisons between continuous and independent variables were performed using respectively Student t-test, Chi-square test or ANOVA. All statistical analyses were performed using SPSS version 21.0 (IBM, Armonk, New York). *P.* values <0.05 were considered significant.

## Findings

**Fig. 2:**
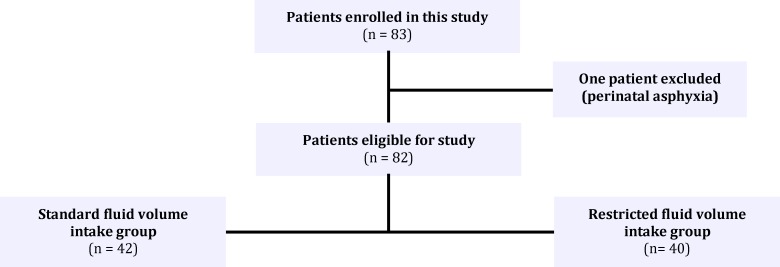
Patients included in this study

Of the 83 neonates enrolled in study, one patient in restricted fluid volume intake group was removed from the study when diagnosed as perinatal asphyxia (Fig. 2). 42 patients were assigned to standard fluid volume intake (SFVI) and 40 patients to restricted fluid volume intake (RFVI) group (Table 1). Contemporary 53 well babies (63.3% males) were studied for weight and urine specific gravity alterations. Birth weights and second day body weight of well babies were 3216 and 3032g, respectively. Weight loss in second day of life was 184.0±160.4 g, in the SFVI group it was 79.8±198.5 g and in RFVI group 86.8±115.1 g, which revealed a significant difference between the weight loss in well baby newborn infants and the two study groups (*P*=0.01,* P*=0.02). No significant difference existed between weight loss in second and third day of life in SFVI and RFVI (Table 2, 3). First day urine specific gravity in the SFVI and RFVI group, and well baby newborns was 1.12±6.5, 1.10±4.6, and 1.11±5.6, respectively. No significant difference was seen in urine specific gravity at first day of life between well baby newborns and SFVI (*P*=0.91) or RFVI group (*P*=1.00). Also no significant difference existed between urine specific gravity in SFVI and RFVI group at first day of life (Table 2, 3). 

 Urine specific gravity in SFVI group of well-baby newborns at the second day of life was 1.14±5.4. There was significant difference between urine specific gravity at the second day of life in well baby newborns and SFVI (*P*<0.001) or RFVI group (*P*<0.001). The difference between urine specific gravity in SFVI and RFVI groups in the second and third day of life was not significant. 

 In the SFVI group 18 cases (42.8%) needed nasal continuous positive airway pressure (NCPAP) and one case (2.38%) mechanical ventilation; also, in RFVI group 13 (32.5%) cases needed NCPAP and 2 (5%) patients mechanical ventilation.

**Table 1:** Patients’ characteristics with diagnosis of transient tachypnea of newborn

**Variable**	**Standard fluid volume**	**Restricted fluid volume**	***P.*** ** value**
**Male sex**	24 (60%)	32 (76.2%)	0.23
**Birth weight gr.**	2806 (360)	2883.5 (542.5)	1.00
**Gestational Age weeks**	36 (1.3)	36 (1.7)	1.00
**Cesarean section**	37 (92.5%)	33 (78.6%)	0.00

 A total of 54.82% of cases in the SFVI group and 62.5% of cases in RFVI group were supported with oxygen hood. A significant difference existed between duration of need for respiratory support (Table 2), however, no significant difference existed in terms of duration of hospitalization between the two groups (*P*= 0.2).

## Discussion

The first main result of this study was that according to urine specific gravity and the degree of weight loss in normal breastfed newborns, in newborns with TTN fluid restriction is safe. It is compatible with the results of Stroustrup et al^[^^16^^]^.

 In the first few days of life urine output of approximately 1 to 3 ml/kg/hour, and urine specific gravity of 1.008 to 1.012 and weight loss of approximately 5% in term infants is in accordance with appropriate fluid and electrolyte balance^[^^22^^]^. In our study weight loss of normal breastfed newborns was 5.7% in the second day of life, while in SFVI it was 2.8% and in the RFVI group 1.9% (the differences are significant). Also the weight loss in third day of life in SFVI was 2.6% and in RFVI group 4.8%, in relation to that of the second day of life, however the difference was not significant. Weight loss in normal breastfed newborns was significantly larger than that in the two studied groups. The difference between urine specific gravity of the two studied groups and breast fed normal newborns in the first day of life was not significant, but the urine specific gravity at the second day of life in breastfed normal newborns was significantly higher than in the two studied groups, while the difference between two studied groups was not significant. Since in our hospital normal newborns are discharged on the second day of life, we could not compare their weight loss and urine specific gravity at the third day of life with the two studied TTN infant groups. Urine specific gravity of the third day of life between SFVI and RFVI groups was not significant. The cause of less weight loss in our patients than in normal breastfed newborns is not clear. It may be due to less volume intake in well baby breastfed infants at the first few days of life. Inappropriate antidiuretic hormone secretion in patients with pulmonary disease is reported^[^^23^^]^, but hyponatremia and elevated urine specific gravity was not detected in any of our patients.

**Table 2:** Patients’ weight and urine specific gravity alterations

**Variable**		**Standard fluid volume**	**Restricted fluid volume**	***P.*** ** value**
**Weight (gr.)**	**First day**	2806 (±360)	2883.5 (±542.5)	1
	**Second day**	2726.2 (±331)	2828.3 (±591.9)	1
	**Third day**	2654.5 (±348)	2744.5 (±574.2)	0.4
**Urine specific gravity**	**First day**	1.12 (±6.5)	1.10 (±4.6)	0.7
	**Second day**	1.09 (±4.3)	1.09 (±4)	1
	**Third day**	1.09 (±3.6)	1.08 (±3.3)	0.4
**Respiratory support (hour)**	**Oxygen **	35.4 (±3502)	33.4 (±26.2)	0.004
	**NCPAP **	66.6 (±41.2)	27.8 (±15)	0.003
	**MV **	38	26.0 (±2.8)	0.2
**Hospitalization (day)**		6.7(2.)	6.1(2.2)	0.2

NCPAP: Nasal Continuous Positive Airway Pressure; MV: Mechanical Ventilation

**Table 3:** Alteration of weight and urine specific gravity in studied groups

**Parameter**		**Mean**	**Std deviation**	**Minimum**	**Maximum**
**Frist day weight (gr)**	**RFVG **	2883.6	542.0	2100	4200
	**SFVG**	2806.0	360.9	2200	3750
	**Normal G**	3216.0	525.4	2100	4500
**Second day weight (gr)**	**RFVG**	2828.4	591.9	1900	4150
	**SFVG**	2726.3	331.1	2100	3600
	**Normal G**	3032.1	460.8	2000	4200
**First day urine SG**	**RFVG**	1.010	4.6	1.004	1.025
	**SFVG**	1.012	6.5	1.003	1.032
	**Normal G **	1.011	5.6	1.003	1.026
**Second day urine SG**	**RFVG**	1.009	4	1.003	1.024
	**SFVG**	1.009	4.3	1.003	1.024
	**Normal G**	1.014	5.4	1.003	1.032

SG: Specific Gravity; RFVG: restricted fluid volume intake group, SFVG: standard fluid volume intake group

Based on this finding, we suspected that restricted fluid volume strategy and even lower volume may be safe in fluid management of TTN.

 The second main finding of this study was a significant difference between number of cases needing NCPAP in restricted fluid volume intake group (13.2% ) in comparison with SFVI group (42.8%) and also between duration of need for NCPAP and oxygen (*P*=0.003 and 0.004), but the difference between the cases needing mechanical ventilation was not significant. Strustrup et al^[^^16^^]^ found no difference of respiratory support duration between SFVI and RFVI group [*P*=0.209], but in cases with severe TTN (respiratory support ≥48hours) the duration of respiratory support was significantly shorter in RFVI than in SFVI group (*P*=0.048). The effect of fluid restriction on decreasing need to NCPAP in neonate with TTN is not clear. Lewis et al^[^^24^^]^ reported that weight loss in the first day of life associated with furosemide administration to newborns with TTN was not accompanied by differences in TTN severity or length of the symptoms. The third finding of our study revealed that duration of hospital stay between the two studied groups was not significant which may be related to the delay in receiving full enteral feeding and hyperbilirubinemia.

 Randomization was not considered in this survey and this is the limitation of our study. Also this study did not show the minimum safe fluid volume in management of newborns with TTN so that further studies are required to achieve more accurate results.

## Conclusion

Our study showed that limited fluid administered to newborns with transient tachypnea of newborn is safe and resulted in shorter duration of respiratory support.
